# Innovative Approaches in Environmental Medicine: Redox/Detoxification Biomarkers in Environmental Intolerances

**DOI:** 10.1155/2013/691624

**Published:** 2013-12-05

**Authors:** Giuseppe Valacchi, Daniela Caccamo, Edward Pelle, Chiara De Luca

**Affiliations:** ^1^Department of Life Sciences and Biotechnology, University of Ferrara, Via L. Borsari 46, 44121 Ferrara, Italy; ^2^Department of Food and Nutrition, Kyung Hee University, 1 Hoegi-dong, Dongdaemun-gu, Seoul 130-701, Republic of Korea; ^3^Department of Biochemical, Physiological and Nutritional Sciences, Policlinico Universitario, University of Messina, 98122 Messina, Italy; ^4^Skin Biology, The Estee Lauder Companies, Melville, NY 11747, USA; ^5^Environmental Medicine, New York University School of Medicine, New York, NY 10016, USA; ^6^Centre of Innovative Biotechnological Investigations (CIBI-Nanolab), Novoslobodskaya Street 36/1, 127055 Moscow, Russia

The increase of old-and-new pollutants heavily conditions quality of civil and working life. Concern about the exponential increase of both allergic and nonallergic environmental sensitivity-related illnesses (SRI) has brought into focus a new inhomogeneous cluster of adverse—often socially and professionally disabling—clinical multiorgan conditions, still lacking a satisfactory nosologic classification. These comprise the prototypical multiple chemical sensitivity, along with fibromyalgia, chronic fatigue syndrome, sick building/house syndrome, hypersensitivity to electromagnetic fields, and other syndromes, commonly so far considered as “idiopathic” by medical community. They are elicited by exposure to low-to-negligible doses of diverse, multiple, environmental-borne physical, chemical, and biological triggers, innocuous to the general population, that is, xenobiotic chemicals, drugs, and metals, electro-magnetic or nuclear radiations, and new iatrogenic factors like biocompatible implants, specific food, or microbial allergens.

With this special issue we attempted the challenging effort to gather experts in the field as to contribute to the scant but constantly growing laboratory and epidemiologic studies highlighting common molecular features of SRI, on a genetic or metabolic base. The still open issues here addressed were (a) identifying distinctive biologic and clinical effects of different categories of environmental/nutritional stressors that may be relevant to better describe molecular mechanisms and natural history of these emerging disease conditions, (b) searching reliable biomarkers of diagnostic value for thorough disease classification, and (c) evaluating all available evidence-based information to produce suitable clinical protocols applicable for prevention and treatment, in both the civil and working population at risk of environmental hypersensitivities.

Within this frame, the first two papers contributed significant *in vitro* pieces of evidence on the biological efficacy of very low-dose electromagnetic radiation in cell structure and function derangement. E. Calabro' et al. described lipid and protein alterations in SH-SY5Y neuroblastoma cell membranes, with mitochondrial transmembrane potential alterations and significant loss of cell viability, whilst F. Cervellati et al. addressed the often raised issue of teratogenic risks, demonstrating that 17-*β*-estradiol modulates connexins and integrins, and ER-*β* expression induced by high-frequency electromagnetic fields, with hypothesized consequences on trophoblast-derived HTR-8/SVneo cells differentiation and migration. J. V. Gruber and R. Holtz evidenced molecular effects of three commonly used skin chemical lighteners on keratinocytes and melanocytes, especially highlighting the role of ferritin and iron in skin melanization. The following two papers evidenced the role of exogenous nutritional stressors in epigenetic and metabolic alterations leading to obesity. A. Muňoz and M. Costa reported on the updated knowledge about epigenetic changes measurable in both adipocyte and peripheral blood mononuclear cells by chronic inflammatory stimuli deriving from hypercaloric diet and specific prooxidant nutritional components, that may be selectively counteracted by antioxidant-rich foods like Mediterranean or fish/vegetable-based dietetic regimens. The paper by C. Lubrano et al. reviewed the more and more compelling pieces of evidence linking obesity with endocrine disruptors of environmental origin, focusing on the relevance of individually-impaired detoxification of organic chemicals and heavy metals.

In line, the importance of metal sensitivity in multiple chemical sensitivity was addressed by P. Pigatto et al. in the clinical-diagnostic setting, with a study demonstrating the prevalence of mercury traces in biological matrices from subjects with MCS bearing dental amalgams, producing convincing indications on the validity of noninvasive *in vivo *and *ex-vitro *testing of metals for the diagnosis of MCS. A common leading feature of SRI is undoubtedly the severe imbalance in the capability to modulate and detoxify endogenous and environmental-driven free radical formation, accompanied by specific profiles of altered inflammatory cytokines, of detoxifying and antioxidant capacities, and of oxidative stress markers currently under intense observation (i.e., genotoxic aldehydes, 4-hydroxynonenal, isoprostanes, etc.). The Italian group who previously first described a specific combination of metabolic alterations of diagnostic value for multiple chemical sensitivity here contributed, with the paper by P. Caccamo et al., to the strongly debated question of genetic alterations in MCS and fibromyalgia, showing a significant prevalence of gene mutations of phase I metabolizing genes and environmental-sensing receptors in patients suffering MCS, fibromyalgia, and/or chronic fatigue syndrome symptoms, which is possibly also useful for differential diagnosis among SRIs.

Hopefully, the results presented in this issue concerning the complex, uncompleted SRI models, which stand far away from classical toxicology approaches, will have contributed innovative solutions applicable to environmental toxicology and medicine, through modern comprehensive protocols of genomic, epigenomic, and metabolomic diagnostics, complying to good practice criteria, validated for clinical use. In association with toxico- and pharmacogenomics, these are bound to offer a solid rationale for still-to-come, evidence-based individualized therapy of SRI based on antioxidant/chelator/natural immunomodulating treatments.

Papers gathered in the issue will hopefully stimulate the ongoing efforts to identify specific biomarkers of environmental hypersensitivity to be measured in the clinical setting, especially applicable for professionally borne environmental-connected disorders, which are here extensively approached in the paper by A. Martini et al. They may in perspective also provide mechanistic insights, prognostic and therapeutic indicators, and nutritional/lifestyle recommendations for other recognized, difficult-to-cure, and chronic inflammatory conditions with suspected environmental-borne etiological cofactors, like atopic or autoimmune skin pathologies or metabolism diseases.

*Giuseppe Valacchi* *Giuseppe Valacchi* *Daniela Caccamo* *Daniela Caccamo* *Edward Pelle* *Edward Pelle* *Chiara De Luca* *Chiara De Luca*

